# Post‐treatment urinary sarcosine as a predictor of recurrent relapses in patients with prostate cancer

**DOI:** 10.1002/cam4.1767

**Published:** 2018-09-12

**Authors:** Jaromir Gumulec, Martina Raudenska, Dalibor Pacik, Mariana Plevova, Alena Sorokac‐Kubolkova, Zuzana Lackova, Natalia Cernei, Vladislav Strmiska, Ondrej Zitka, Zbynek Heger, Vojtech Adam

**Affiliations:** ^1^ Department of Pathological Physiology Faculty of Medicine Masaryk University Brno Czech Republic; ^2^ Department of Urology University Hospital Brno Faculty of Medicine Masaryk University Brno Czech Republic; ^3^ Faculty of Medicine Masaryk University Brno Czech Republic; ^4^ Department of Chemistry and Biochemistry Mendel University in Brno Brno Czech Republic; ^5^ Central European Institute of Technology Brno University of Technology Brno Czech Republic

**Keywords:** outcome, prostate cancer, relapse, sarcosine, survival

## Abstract

To date, there has been no evidence regarding the association between urinary sarcosine content and prostate cancer survival. Our main objective was to investigate whether levels of post‐treatment urinary sarcosine are associated with relapse. The inclusion criteria were (in accordance with EAU 2017) as follows: histopathologically verified adenocarcinoma in prostate biopsy cores or specimens from transurethral resection of the prostate (TURP) or prostatectomy for benign prostatic enlargement (BPE) with retained ability to urinate. The median follow‐up was 53 months. In the study, we retrospectively evaluated a cohort of 511 patients with prostate cancer with various risk factors and treatment strategies. Post‐treatment sarcosine levels were elevated in 266 (52%) patients and highly elevated (≥200 nmol/L) in 71 (13%) patients. Urinary sarcosine content was significantly associated with number of relapses that patients experienced, *P* = 0.002 for sarcosine ≥200 vs ≤30 nmol/L. Multivariate analysis revealed that sarcosine was an independent predictor of recurrent relapses (≥2 relapses with an intermediate period of remission), HR = 3.89 (95% CI 1.29‐11.7) for sarcosine >200 vs <30 nmol/L. This trend was even more pronounced in a subgroup of patients who underwent radical prostatectomy, HR = 3.29 (95% CI 1.06‐10.18), where (single) relapse‐free survival could also be predicted by sarcosine levels, HR = 1.96 (1.05‐3.66). Urinary sarcosine may become a possible predictor for patients’ outcomes, because patients with elevated post‐treatment sarcosine could be predicted to have recurrent relapses of the disease.

## 1 INTRODUCTION

Prostate cancer is one of the most frequently diagnosed nonskin solid cancers and is included among the main causes of cancer deaths in numerous developed countries.^1^, ^2^ The use of prostate‐specific antigen (PSA) levels has led to important progress in early diagnostics. Approximately 85% of patients are now diagnosed with localized disease.^2^ When PSA levels rise to a certain threshold after prostate cancer treatment, the patient displays the so‐called biochemical recurrence (BCR). Nevertheless, not all patients with BCR have the same prognosis, and classification of patients into appropriate risk groups is important and indispensable.^3^ Unfortunately, the best existing markers cannot exactly identify a poor‐prognosis group of patients who eventually would fail therapy and would display recurrence of the disease.

Metabolomic profiling of prostate cancer revealed elevated levels of sarcosine (*N*‐methyl glycine) nominating this amino acid as an oncometabolite. Significant elevation of sarcosine was also detectable in the urine of prostate cancer patients.^4^, ^5^ Prostate cancer, like many tumors, is known to exhibit a “field cancerization effect” that influences the metabolism of adjacent benign tissues.^4^, ^6^ The levels of sarcosine in tumor‐adjacent benign tissues could be potentially influenced by the presence of a tumor, as increased sarcosine production in tumor‐adjacent tissue could support the growth of tumor cells and consequently lead to triggering of a recurrence of the disease.^5^, ^7^ Therefore, the aim of the present study was to investigate the potential of urinary sarcosine as a marker of oncologic outcome (relapse) in prostate cancer patients of various subgroups [castration‐resistant prostate cancer (CRPC) patients who underwent radical prostatectomy, radiotherapy, etc.].

## 2 MATERIALS AND METHODS

### 2.1 Patients

This study was approved by the University Hospital Brno Ethics Committee (No. 458712, Brno, Czech Republic), and informed consent was obtained from all subjects. A total of 511 patients from the Urology Clinic, University Hospital Brno with diagnosed prostate cancer were selected for the study. The inclusion criteria were (in accordance with EAU 2017) as follows: histopathologically verified adenocarcinoma in prostate biopsy cores or specimens from transurethral resection of the prostate (TURP) or prostatectomy for benign prostatic enlargement (BPE) with retained ability to urinate. The exclusion criteria involved the following: not retained ability to urinate, urinary catheter, epicystostomy, nephrostomy, or patients with histopathologically verified cancer after radical cystectomy with construction of Bricker bladder. Treatment was performed in accordance with EAU 2017 guidelines.^8^ “Mid‐stream” urine samples in a sterile container were collected from each patient with histologically verified prostate cancer, except those mentioned above, who were excluded. Urine samples were fresh (not stored in the fridge) and immediately analyzed.

### 2.2 Detection of sarcosine

Prior to sarcosine determination, urine was diluted with sterile MiliQ water (1:1) and analyzed using a high‐performance liquid chromatography (HPLC) HP 1100 Series (Palo Alto, CA, USA). The column effluent was monitored with a diode‐array detector at *λ* 338 nm and *λ* 262 nm and a fluorescence detector (*λ*_exc_ = 350 nm, *λ*_em_ = 450 nm) using *o*‐phthalaldehyde and fluorenylmethyloxycarbonyl chloride reagents for precolumn derivatization. Separation was carried out on a Zorbax Eclipse AAA column with dimensions of 150 × 4.6 nm and a particle size of 3.5 μm (Agilent Technologies, Santa Clara, CA, USA). The compounds were eluted with a linear upward gradient of mobile phases composed by acetonitrile/water (90:10 *v/v*) and 0.1 M ammonium formate in water. Determined sarcosine levels were related to urinary creatinine that was analyzed on a BS‐400 automated spectrophotometer (Mindray, Shenzhen, China) using a commercial kit (Greiner, Stuttgart, Germany) according to the manufacturer's instructions.

Cutoff values of urinary sarcosine were determined using the minimum p value approach and rounded to clinically relevant (convenient) values. Concentrations <30 nmol/L were considered low based on ROC analysis between the control cohort and the cohort of patients with diagnosed cancer (determined cutoff = 29.51 nmol/L). Moreover, this cutoff corresponded to the 1st tercile of sarcosine concentrations (32.35 nmol/L). Another cutoff point, 200 nmol/L, discriminated between good and poor relapse‐free survival while maintaining sufficient numbers of patients in groups.

### 2.3 Statistical treatment of data

Patients were generally followed once every 3‐6 months. Follow‐up consisted of history, physical examination, routine blood tests (include PSA level) and urine sampling, and ultrasound examination. In indicated cases, a bone scan was provided.

Disease recurrence was defined by two consecutive PSA values of >0.2 ng/mL and rising for radical prostatectomy or any PSA increase >2 ng/mL higher than the PSA nadir value, regardless of the serum concentration of the nadir for radiotherapy (RTOG‐ASTRO Phoenix Consensus).

The categorical and continuous variables depending on sarcosine level groups were analyzed using a *χ* ^2^ test or Mann‐Whitney *U* test, respectively. Survival analysis was performed using the Kaplan‐Meier approach, followed by the log‐rank test. Association between clinicopathological characteristics, including sarcosine level, Gleason grade, tumor staging, PSA levels, and patient's outcome, was determined in multivariate models using the Cox proportional hazard regression. The *P* values <0.05 was considered significant, unless noted otherwise. Analyses were performed using Statistica 12.2 (Dell, OK, USA) and easyROC 1.3 (http://www.biosoft.hacettepe.edu.tr/easyROC/).

## 3 RESULTS

### 3.1 Characterization of patients

A total of 511 patients with histologically verified (prostatectomy, TURP, or biopsy) acinar adenocarcinoma were included in the follow‐up. Of those, 346 underwent radiotherapy and 46 underwent surgery as a primary therapy strategy, 85 received hormonal therapy only, and 34 were included in an active surveillance group. The length of the follow‐up ranged from 0 to 293 months with a median of 53 months. Sarcosine levels were determined during the last 2 years of the follow‐up.

The median age of all 511 patients in the prostate cancer cohort was 75 years (IQR: 69‐79). Table 1 shows the clinicopathological characteristics of prostate cancer patients and their association with urinary sarcosine level. Patients with urinary sarcosine >200 nmol/L (compared to groups with lower sarcosine) had worse prognostic indicators, including castration‐resistant status (*P* = 0.013), a higher number of relapses (*P* = 0.012), higher PSA (<0.001), and metastatic dissemination (*P* = 0.007). Similarly, patients with urinary sarcosine between 30 and 200 nmol/L tended to have more frequent lymph node positivity compared to those with sarcosine <30 nmol/L. With regard to CRPC phenotype, urinary post‐treatment sarcosine levels were 41.0 (IQR 21.0‐98.0) and 87.0 (IQR 22.5‐533.9) nmol/L for CRPC‐negative and CRPC‐positive cases, respectively. Regarding number of relapses, sarcosine levels were 42.0 (IQR 21.0‐98.0), 35.5 (IQR 18.0‐79.0), and 136.0 (IQR 34.0‐740.0) nmol/L for 0, 1, and ≥2 relapses, respectively.

**Table 1:** Baseline clinicopathological characteristics of 511 patients with prostate cancer according to post‐treatment sarcosine level

Characteristic	All patients	Post‐treatment level of urinary sarcosine (nmol/L)			*P* value		
	(n = 511)	≤30	30‐200	≥200	30‐200	≥200	≥200
		n = 174 (34%)	n = 266 (52%)	n = 71 (13%)	vs ≤30	vs ≤30	vs 30‐200
Age at diagnosis, y	—	—	—	—	0.392	0.715	0.758
Median	75	74	75	75	—	—	—
IQR	69‐79	69‐80	69‐80	69‐78	—	—	—
PSA, ng/mL	—	—	—	—	0.977	0.000	0.000
Median	8.0	7.8	7.7	12.2	—	—	—
IQR	4.8‐16.1	4.8‐14.0	4.4‐15.0	6.0‐49.4	—	—	—
Gleason grade, no (%)	—	—	—	—	0.209	0.576	0.243
≤6	226 (44%)	70 (14%)	124 (24%)	32 (6%)	—	—	—
7	153 (30%)	53 (10%)	83 (16%)	17 (3%)	—	—	—
≥8	132 (26%)	51 (10%)	59 (12%)	22 (4%)	—	—	—
Clinical T stage, no. (%)	—	—	—	—	0.013	0.503	0.014
T1	199 (39%)	67 (13%)	110 (22%)	22 (4%)	—	—	—
T2	156 (31%)	45 (9%)	90 (18%)	21 (4%)	—	—	—
T3	146 (29%)	60 (12%)	58 (11%)	28 (5%)	—	—	—
n.s.	10 (2%)	2 (<1%)	8 (2%)	—	—	—	—
Lymph node positivity, no. (%)	—	—	—	—	0.033	0.263	1.000
cN+	3 (1%)	3 (1%)	0	0	—	—	—
cNx or cN0	498 (97%)	169 (33%)	258 (50%)	71 (14%)	—	—	—
n.s.	10 (2%)	2 (<1%)	8 (2%)	0	—	—	—
Metastasis positivity, no. (%)	—	—	—	—	0.109	0.248	0.007
cM+	19 (4%)	8 (2%)	5 (1%)	6 (1%)	—	—	—
cMx or cM0	481 (94%)	164 (32%)	252 (49%)	65 (13%)	—	—	—
n.s.	11 (2%)	2 (<1%)	9 (2%)	0	—	—	—
Therapy strategy, no. (%)	—	—	—	—	0.001	0.016	0.002
Surgery	46 (9%)	17 (3%)	15 (3%)	14 (3%)	—	—	—
Radiotherapy	346 (68%)	133 (26%)	173 (34%)	40 (8%)	—	—	—
Only hormonal therapy	85 (17%)	20 (4%)	52 (10%)	13 (3%)	—	—	—
Active surveillance	34 (7%)	4 (1%)	26 (5%)	4 (1%)	—	—	—
Castration‐resistant, no. (%)	—	—	—	—	0.566	0.013	0.001
No	487 (95%)	167 (33%)	258 (50%)	62 (12%)	—	—	—
Yes	24 (5%)	7 (1%)	8 (2%)	9 (2%)	—	—	—

IQR, interquartile range.

### 3.2 Characterization of controls

A total of 37 healthy male individuals were included, with a median age of 58 (IQR 55‐56), in order to define the cutoff between healthy individuals and patients with diagnosed cancer. Patients had significantly higher urinary sarcosine compared to healthy individuals, 42.0 nmol/L (IQR 21.0‐100.0) vs 9.0 nmol/L (IQR 1.0‐42.0), *P* < 0.001. Using ROC, sensitivity 66.7% and specificity was determined as 67.6% for a cutoff of 30 nmol/L with AUC = 0.73 (95% CI 0.63‐0.83). Table 2 shows clinicopathological characteristics of healthy subjects involved in the study.

**Table 2:** Baseline clinicopathological characteristics of control subjects and comparison with cohort

Characteristic	Controls	Level of urinary sarcosine (nmol/L)			*P* value	
	(n = 37)	≤30	30‐200	≥200	30‐200	Cohort
		n = 25 (68%)	n = 11 (30%)	n = 1 (3%)	vs ≤30	vs control
Urinary sarcosine, nmol/L			—	<0.001	—	<0.001
Age at diagnosis, y	—	—	—	—	0.877	<0.001
Median	58	58	60	30	—	—
IQR	55‐65	55‐65	56‐65	—	—	—
PSA, ng/mL				0.513	<0.513	<0.001
Median	2.8	2.9	2.1	0.3	—	—
IQR	0.9‐4.9	0.9‐4.9	0.9‐4.1	—	—	—

IQR, interquartile range.

### 3.3 Recurrence‐free survival

During the follow‐up, 88 patients (17.2%) experienced biochemical recurrence. Of 88 patients, 22 patients (4%) developed recurrent relapses (≥2 relapses with an intermediate period of biochemical remission). Of 88, five developed local recurrence and seven developed metastatic recurrences (characterization of these subjects is presented in Table 3). Patients with urinary sarcosine levels of 30‐200 nmol/L had a longer period between diagnosis and recurrence compared to the group of patients with <30 nmol/L (*P* = 0.04).

**Table 3:** Characteristics of 88 patients who experienced disease recurrence according to urinary sarcosine levels

Characteristic	All patients	Post‐treatment level of urinary sarcosine (nmol/L)			*P* value		
	(n = 88)	≤30	30‐200	≥200	30‐200	≥200	≥200
		n = 29 (33%)	n = 40 (45%)	n = 19 (22%)	vs ≤30	vs ≤30	vs 30‐200
Interval between diagnosis and recurrence	—	—	—	—	0.035	0.916	0.080
Median, y	5	4.4	6.5	3.2	—	—	—
IQR	2.6‐8.2	2.0‐6.4	3.0‐9.2	2.4‐7.4	—	—	—
Number of relapses, no (%)	—	—	—	—	0.66	0.012	0.002
0	423 (83%)	144 (28%)	227 (44%)	52 (10%)	—	—	—
1	66 (13%)	25 (5%)	32 (6%)	9 (2%)	—	—	—
2‐3	15 (3%)	3 (1%)	6 (1%)	6 (1%)	—	—	—
>3	7 (1%)	2 (<1%)	1 (<1%)	4 (1%)	—	—	—
Stage at disease recurrence, no. (%)							
Local recurrence	5 (1%)	2 (<1%)	3 (1%)	0	0.148	—	—
Metastatic disease	7 (1%)	2 (<1%)	3 (1%)	2 (<1%)	0.148	0.245	0.148

IQR, interquartile range.

**Figure 1:**
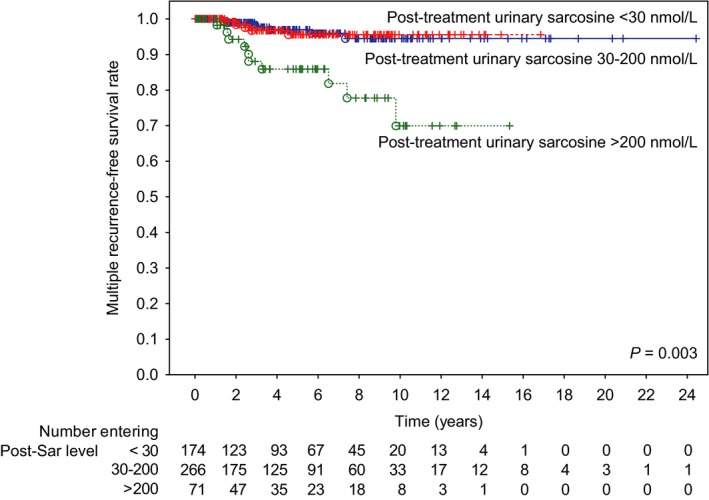
Recurrence‐free survival for urinary sarcosine levels <30 nmol/L and between 20 and 200 nmol/L vs >200 nmol/L post‐treatment

Table 4 demonstrates that patients in the post‐treatment urinary sarcosine group >200 nmol/L could not be predicted to have a risk of disease recurrence. However, multiple recurrences (≥2 relapses) could be predicted by a group of patients with sarcosine levels of >200 nmol/L, with a five‐year recurrence‐free survival rate of 78.0% in patients with >200, 91.5% in patients with 30‐200, and 86.2% in patients with <30 nmol/L (*P* = 0.003, see Figure 1). Hence, there was a distinct association between post‐treatment urinary sarcosine levels and ≥2 relapses, suggesting that patients with high urinary sarcosine had a higher risk of recurrent relapses. Moreover, apart from sarcosine, univariate and multivariate tests were performed to determine predictors of patients’ outcomes. A stepwise model of multivariate analysis showed that PSA group and Gleason grade were independent risk factors for the recurrence of the disease for both single and recurrent relapses.

**Table 4:** Risk factors for predicting multiple disease recurrence and recurrence‐free survival in 511 patients

	RFS, ≥2 relapses			RFS		
	Univariate	Multivariate		Univariate	Multivariate	
	*P* value	HR (95% CI)	*P* value	*P* value	HR (95% CI)	*P* value
Post‐treatment urinary sarcosine, nmol/L	—	—	—	—	—	—
30‐200 vs < 30	0.996	1.09 (0.34‐3.45)	0.206	0.452	—	—
>200 vs <30	0.001	3.89 (1.29‐11.7)	0.003	0.077	—	—
Age, y	—	—	—	—	—	—
Gleason grade	—	—	—	—	—	—
>7 vs <7	0.008	2.44 (0.94‐6.36)	0.017	0.000	1.85 (1.12‐3.08)	0.00
7 vs <7	0.690	0.64 (0.17‐2.45)	0.153	0.190	0.58 (0.30‐1.13)	0.01
Clinical T stage	—	—	—	—	—	—
cT3 vs cT1	0.017	—	—	0.127	—	—
cT2 vs cT1	0.172	—	—	0.194	—	—
PSA, ng/mL	—	—	—	—	—	—
10‐20 vs <10	0.014	3.67 (0.91‐14.82)	0.360	0.023	1.71 (0.98‐2.98)	0.49
>20 vs <10	0.000	5.18 (1.41‐19.07)	0.036	<0.001	2.11 (1.24‐3.57)	0.04
Lymph node positivity (cN+ vs cNx or cN0)	0.882	N/A	N/A	<0.001	N/A	N/A
Metastasis positivity (cM+ vs cMx or cM0)	0.003	N/A	N/A	<0.001	N/A	N/A
Castration‐resistant (yes vs no)	<0.001	N/A	N/A	<0.001	N/A	N/A

“—” indicates parameter not selected in Cox regression, CI, confidence interval; ; HR, hazard ratio; N/A, not applicable; RFS, recurrence‐free survival.

Using univariate testing, it was revealed that lymph node positivity, metastatic positivity, and castration‐resistant status were also strongly associated with RFS, but due to the limited number of patients in these subgroups, those three clinicopathological factors were not included in a multivariate model.

Further, the association of disease recurrence was analyzed in a subgroup of patients who underwent either radiotherapy or surgical treatment despite the fact that the sarcosine levels were affected by the therapeutic strategy. Table 5 summarizes the survival analysis of this subgroup. Trends were similar to those in a previous analysis, and (single) recurrence may also be predicted by urinary sarcosine >200 nmol/L using either a univariate or multivariate model (*P* = 0.01).

**Table 5:** Risk factors for predicting multiple disease recurrence and recurrence‐free survival in a subgroup of patients who underwent surgery (46 subjects) and radiotherapy (346 subjects)

	RFS, ≥2 relapses			RFS		
	Univariate	Multivariate		Univariate	Multivariate	
	*P* value	HR (95% CI)	*P* value	*P* value	HR (95% CI)	*P* value
Post‐treatment urinary sarcosine, nmol/L	—	—	—	—	—	—
30‐200 vs <30	0.881	0.83 (0.25‐2.74)	0.119	0.699	0.92 (0.53‐1.59)	0.09
>200 vs <30	0.001	3.29 (1.06‐10.18)	0.006	0.032	1.96 (1.05‐3.66)	0.01
Age, y	—	—	—	—	—	—
Gleason grade	—	—	—	—	—	—
>7 vs <7	0.032	—	—	0.031	2.03 (1.18‐3.5)	0.00
7 vs <7	0.679	—	—	0.247	0.63 (0.31‐1.28)	0.09
Clinical T stage	—	—	—	—	—	—
cT3 vs cT1	0.073	—	—	0.405	—	—
cT2 vs cT1	0.221	—	—	0.274	—	—
PSA, ng/ml	—	—	—	—	—	—
10‐20 vs <10	0.051	3.3 (0.78‐13.89)	0.515	0.051	—	—
>20 vs <10	<0.001	5.28 (1.43‐19.42)	0.028	<0.001	—	—
Lymph node positivity (cN+ vs cNx or cN0)	0.888	N/A	N/A	<0.001	N/A	N/A
Metastasis positivity (cM+ vs cMx or cM0)	0.007	N/A	N/A	<0.001	N/A	N/A
Castration‐resistant (yes vs no)	<0.001	N/A	N/A	<0.001	N/A	N/A

“—” indicates parameter not selected in Cox regression, N/A, not applicable; RFS, recurrence‐free survival; HR, hazard ration, CI, confidence interval.

Although the stepwise model demonstrates that sarcosine is a RFS predictor independent from PSA, combinations of PSA (<10, 10‐20, and >20 ng/mL) and urine sarcosine groups (<30, 30‐200, and >200 nmol/L) were analyzed for RFS prediction (Table 6). Weak but significant correlation between PSA and urine sarcosine was observed (Spearman *R* = 0.28, *P* < 0.001). It was found out that the highest hazard ratio value occurs in patients with simultaneous high PSA and high urinary sarcosine, HR = 4.36 (1.75‐10.85), *P* = 0.002. Such high HR was not determined either in any other >20 nmol/L PSA group in this analysis. Similar analysis was also performed for the combination of Gleason/Sarcosine. Nevertheless, due to numbers of patients in individual groups (five patients in low grade/high sarcosine), it was not possible to analyze it reliably, so it was not possible to take into account other potentially confounding clinicopathological factors and to analyze multiple relapses in a similar manner.

**Table 6:** Combination of PSA/Gleason/urine sarcosine for predicting recurrence‐free survival

Comparison	Group	N patients	RFS, multivariate	
			HR (95% CI)	*P* value
PSA (ng/mL)/urinary sarcosine (nmol/L)	<10/<30 (reference)	8 (9%)	1.00	N/A
	<10/30‐200	16 (18%)	1.30 (0.55‐3.10)	0.549
	10‐20/30‐200	10 (11%)	1.44 (0.56‐3.70)	0.448
	<10/>200	4 (5%)	2.03 (0.61‐6.76)	0.247
	>20/30‐200	14 (16%)	2.55 (1.06‐6.13)	0.036
	>20/<30	9 (10%)	3.05 (1.17‐7.92)	0.022
	10‐20/<30	12 (14%)	3.32 (1.36‐8.14)	0.009
	10‐20/>200	4 (5%)	3.56 (1.06‐11.89)	0.039
	>20/>200	11 (13%)	4.36 (1.75‐10.85)	0.002
Grading (Gleason score)/urinary sarcosine (nmol/L)	<7/<30	8 (9%)	1.00	N/A
	<7/30‐200	9 (10%)	0.48 (0.19‐1.25)	0.135
	>7/<30	21 (24%)	1.25 (0.55‐2.83)	0.593
	>7/30‐200	31 (35%)	1.41 (0.64‐3.13)	0.397
	<7/>200	5 (6%)	1.71 (0.56‐5.25)	0.345
	>7/>200	14 (16%)	2.11 (0.88‐5.04)	0.094

Group with <10 ng/mL PSA, Gleason <7 and <30 nmol/L sarcosine used as reference for others. CI, confidence interval; HR, hazard ratio; RFS, recurrence‐free survival.

### 3.4 Impact of post‐relapse sarcosine normalization

In the next step, we determined how the chronology of relapse and sarcosine measurement affected its values. In patients who underwent relapse, urinary sarcosine was determined in a range of 11 months before relapse to 196 months after relapse. There was no significant association between pre‐relapse and post‐relapse urinary sarcosine concentrations (*P* = 0.68) as determined by paired test. Moreover, it was determined that there was no correlation between time related to relapse and sarcosine concentrations (Spearman *R* = −0.06, *P* = 0.61). These findings suggest that urinary sarcosine was not affected at the time‐point when it was determined, whether it was before or after the actual relapse.

## 4 DISCUSSION

To the best of our knowledge, this is the first report documenting the prognostic significance of urinary sarcosine in prostate cancer. The clinical value of sarcosine quantitation has been investigated in some studies, suggesting that there is a promising association between sarcosine elevation and cancer progression.^4^, ^9^, ^10^, ^11^ Nevertheless, some studies oppose these results and note limited clinical potential of sarcosine.^12^, ^13^ We anticipate that these contradictory results could be caused by several factors, including conditions used for the treatment of samples and detection of sarcosine, as well as concepts of the studies themselves. Regarding the analytical conditions, including sample treatment and sarcosine detection, we previously revealed that biochemical stability of sarcosine in urinary specimens stored in a deep freezer is quite low and results in a decrease in sarcosine amount.^14^ Therefore, we avoided any complex sample preparation and performed analyses directly after urine dilution in this study, which significantly contributed to the simplicity of the analysis. Another important point is that the studies often use gas chromatography with mass detection, which, however, lacks specificity for the analysis of complex biological matrices.^15^ This is compensated by demanding sample preparations, such as solid‐phase or liquid‐liquid extractions,^4^, ^13^ during which sarcosine can be continuously lost resulting in false‐negative results. Quantitation of sarcosine is further complicated due to its identical molecular weight with isomer alanine (89.0932 Da). This phenomenon markedly complicates their distinguishing using mass spectrometry and can result in a quantitation of a mixture of sarcosine and alanine rather than sarcosine alone as mentioned by Meyer et al.^16^

In the present study, we retrospectively evaluated a cohort of 511 patients with prostate cancer with various risk factors and treatment strategies and analyzed the impact of urinary sarcosine levels on their outcomes. In order to define the cutoff values for urinary sarcosine, a control cohort was employed. Although there was a significant difference between the control and cancer groups with defined sensitivity and specificity, this fact was not further analyzed because of several limitations. First, the control cohort was of significantly lower age; second, the control cohort sample was small (37 men); third, the control subjects did not undergo identical diagnostic procedures to the patients in the cancer cohort. However, we focused on other outcomes, including relapses, and found that patients with high post‐treatment urinary sarcosine levels >200 nmol/L had a significantly higher risk of recurrent relapses compared to patients with relatively low sarcosine levels <30 nmol/L. Moreover, in a subgroup of patients who underwent radical prostatectomy or radiotherapy, high urinary sarcosine represented a significant risk predictor for even (one) recurrence. One possible reason for this phenomenon is that high post‐treatment urinary sarcosine reflects changes in tumor‐adjacent tissue and evolving tumor‐supporting stroma rather than any inherent metastatic potential of cancer cells themselves. Intensive sarcosine production in tumor‐adjacent tissue could support growth and invasion of tumor cells that survived therapy 5, 7, 17 and consequently could lead to triggering of a recurrence of the cancer disease.

Our study has some important limitations. First, it was retrospective, and our group of patients was quite heterogeneous, mainly due to the variable follow‐up schedule (patients without relapse may have had a relapse shortly after sarcosine sampling). Second, the number of patients in some groups was too low (node positivity, metastatic positivity, CRPC status) for them to be included in a multivariate model, but they have the potential to be studied further. Thus, the clinical usefulness of sarcosine quantitation would require further validation, such as a future prospective study with more frequent sarcosine sampling in shorter periods.

This retrospective study dealing with urinary sarcosine in prostate cancer subjects showed that patients with high post‐treatment urinary sarcosine levels had a significantly higher risk of single or, particularly, recurrent relapses compared to patients with relatively low sarcosine levels. Patients with highly elevated sarcosine levels could be predicted to display disease recurrence. Therefore, we suggest that urinary sarcosine may become a promising biomarker of prostate cancer recurrence, especially because of the low cost and noninvasiveness of urinary sarcosine assay.

## CONFLICT OF INTEREST

The authors report no conflict of interest.

## References

[1] Haas GP , Delongchamps N , Brawley OW , Wang CY , de La Roza G . The worldwide epidemiology of prostate cancer: perspectives from autopsy studies. Can J Urol. 2008;15(1):3866‐3871. 18304396 PMC2706483

[2] Jemal A , Siegel R , Ward E , et al. Cancer statistics, 2006. CA Cancer J Clin. 2006;56(2):106‐130. 16514137 10.3322/canjclin.56.2.106

[3] Paller CJ , Antonarakis ES . Management of biochemically recurrent prostate cancer after local therapy: evolving standards of care and new directions. Clin Adv Hematol Oncol. 2013;11(1):14‐23. 23416859 PMC3624708

[4] Sreekumar A , Poisson LM , Rajendiran TM , et al. Metabolomic profiles delineate potential role for sarcosine in prostate cancer progression. Nature. 2009;457(7231):910‐914. 19212411 10.1038/nature07762 PMC2724746

[5] Khan AP , Rajendiran TM , Ateeq B , et al. The role of sarcosine metabolism in prostate cancer progression. Neoplasia. 2013;15(5):491‐501. 23633921 10.1593/neo.13314 PMC3638352

[6] Risk MC , Knudsen BS , Coleman I , et al. Differential gene expression in benign prostate epithelium of men with and without prostate cancer: evidence for a prostate cancer field effect. Clin Cancer Res. 2010;16(22):5414‐5423. 20935156 10.1158/1078-0432.CCR-10-0272 PMC2992073

[7] Heger Z , Gumulec J , Cernei N , et al. Relation of exposure to amino acids involved in sarcosine metabolic pathway on behavior of non‐tumor and malignant prostatic cell lines. Prostate. 2016;76(7):679‐690. 26847870 10.1002/pros.23159

[8] Mottet N , Bellmunt J , Bolla M , et al. EAU‐ESTRO‐SIOG guidelines on prostate cancer. Part 1: screening, diagnosis, and local treatment with curative intent. Eur Urol. 2017;71(4):618‐629. 27568654 10.1016/j.eururo.2016.08.003

[9] Lucarelli G , Fanelli M , Larocca AMV , et al. Serum sarcosine increases the accuracy of prostate cancer detection in patients with total serum PSA less than 4.0?ng/ml. Prostate. 2012;72(15):1611‐1621. 22430630 10.1002/pros.22514

[10] Couzin J . BIOMARKERS metabolite in urine may point to high‐risk prostate cancer. Science. 2009;323(5916):865. 19213886 10.1126/science.323.5916.865a

[11] Koutros S , Meyer TE , Fox SD , et al. Prospective evaluation of serum sarcosine and risk of prostate cancer in the prostate, lung, colorectal and ovarian cancer screening trial. Carcinogenesis. 2013;34(10):2281‐2285. 23698636 10.1093/carcin/bgt176 PMC3786375

[12] Wu H , Liu TT , Ma CG , et al. GC/MS‐based metabolomic approach to validate the role of urinary sarcosine and target biomarkers for human prostate cancer by microwave‐assisted derivatization. Anal Bioanal Chem. 2011;401(2):635‐646. 21626193 10.1007/s00216-011-5098-9

[13] Jentzmik F , Stephan C , Miller K , et al. Sarcosine in urine after digital rectal examination fails as a marker in prostate cancer detection and identification of aggressive tumours. Eur Urol. 2010;58(1):12‐18. 20117878 10.1016/j.eururo.2010.01.035

[14] Heger Z , Cernei N , Krizkova S , et al. Paramagnetic nanoparticles as a platform for FRET‐based sarcosine picomolar detection. Sci Rep. 2015;5:1‐7. 10.1038/srep08868 PMC4352859 25746688

[15] Dunn WB , Ellis DI . Metabolomics: current analytical platforms and methodologies. TrAC‐Trends Anal Chem. 2005;24(4):285‐294.

[16] Meyer TE , Fox SD , Issaq HJ , et al. A reproducible and high‐throughput HPLC/MS method to separate sarcosine from α‐ and β‐alanine and to quantify sarcosine in human serum and urine. Anal Chem. 2011;83(14):5735‐5740. 21635006 10.1021/ac201003r

[17] Heger Z , Rodrigo MAM , Michalek P , et al. Sarcosine up‐regulates expression of genes involved in cell cycle progression of metastatic models of prostate cancer. PLoS ONE. 2016;11(11):1‐20. 10.1371/journal.pone.0165830 PMC5100880 27824899

